# Development of a legume-enriched feed for treatment of severe acute malnutrition

**DOI:** 10.12688/wellcomeopenres.16771.2

**Published:** 2023-01-27

**Authors:** Kevin Walsh, Gael Delamare de la Villenaise de Chenevarin, Joe McGurk, Kathryn Maitland, Gary Frost

**Affiliations:** 1 Section for Nutrition Research, Department of Medicine,, Imperial College London,, London, W12 ONN, UK; 2 Production and Processing Research Department, Campden BRI Group, Chipping Campden, GL55 6LD, UK; 3 Department of Infectious Disease and Institute of Global Health and Innovation, Division of Medicine, Imperial College London, London, W2 1PG, UK; 4 Clinical, KEMRI Wellcome Trust Research Programme, Kilifi, Kenya, PO Box 230, Kenya

**Keywords:** severe acute malnutrition, undernutrition, legume, microbiome, ready-to-use therapeutic food, resistant starch

## Abstract

**Background:** Outcomes in children hospitalised with severe acute malnutrition (SAM) remain poor. The current milk-based formulations focus on restoring weight-gain but fail to address modification of the integrity of the gut barrier and may exacerbate malabsorption owing to functional lactase, maltase and sucrase deficiency. We hypothesise that nutritional feeds should be designed to promote bacterial diversity and restore gastrointestinal (GI) barrier function.

**Methods:** Our major objective was to develop a lactose-free, fermentable carbohydrate-containing alternative to traditional F75 and F100 formulae for the inpatient treatment of SAM. New target nutritional characteristics were developed and relevant food and infant food specific legislation were reviewed. Suitable certified suppliers of ingredients were identified. Processing and manufacture steps were evaluated and optimised for safety (nutritional, chemical and microbiological), and efficacy at meeting target characteristics (lactose-free, containing resistant starch 0.4-0.5% final product weight).

**Results:** A final validated production process was developed and implemented to produce a novel food product for the inpatient treatment of SAM in children in Africa designed to reduce risk of osmotic diarrhoea and support symbiotic gut microbial populations. The final product matched the macronutrient profile of double-concentrated F100, adhered to all relevant legislation regulating infant foods, was lactose free, and contained 0.6% resistant starch. Chickpeas were selected as the source of resistant starch, since they are widely grown and eaten throughout Africa. Micronutrient content could not be matched in this ready-to-use product, so this was replaced at the point of feeding, as was fluid lost through concentration.

**Conclusions:** The processes and product described illustrate the development steps for a novel nutritional product. The new feed product was ready for evaluation for safety and efficacy in a phase II clinical trial in Ugandan children admitted to hospital with SAM (Modifying Intestinal MicroBiome with Legume-Based feed 2: MIMBLE feed 2 (ISRCTN10309022)).

## Introduction

It is estimated that 45% of all childhood mortality is due to undernutrition ^1^. The latest estimates of child malnutrition have recently (2021) been published in a joint report by UNICEF/ World Health Organization (WHO) and World Back Group (URL: https://www.who.int/publications/i/item/9789240025257) Globally there is estimated to be 45.4 million children with wasted and the largest burden of wasting and severe wasting are being experienced in Asia (31.)9 million and 10.3 million respectively) and sub-Saharan Africa (12.1 million and 3 million respectively). For children with the severest form of malnutrition, who require facility/hospital-based care for nutritional rehabilitation the World Health Organization (WHO) recommends milk-based feeds as the primary treatment for severe acute malnutrition (SAM), so-called F75 (starter) and F100 (catch up), whose names are based in their energy content (kilocalories/100mls). The feeds are formulated as a powder-based feed which are reconstituted with sterile water prior to feeding. In the initial stabilisation phase the main focus is on managing acute medical conditions, and cautious feeding ^2^. The introduction of large amounts of carbohydrate in this stage may result in development of refeeding syndrome, so F75 (so called as it contains 0.75kcal/ml) formula contains only enough to support basic physiological function. Once the child is stabilised, the higher energy F100 formula (1kcal/ml) is introduced to support weight gain.

The recommended ingredients were first published by WHO in 1999 ^2^ and commercial formulations have also been developed (Nutriset, Malaunay, France). However, successful implementation requires prolonged hospitalisation or attendance to a feeding centre (for the child and their carer) as F75/F100 prepared from non-sterile water or not stored correctly poses a high risk of bacterial contamination. To address the issues for famine situations in drought-ridden areas, where children are managed in the community with limited access to clean and safe water for reconstitution, ready-to-use therapeutic foods (RUTF) were developed by Valid International, equivalent to F100 in nutritional profile and containing peanuts, milk powder, sugar, oil and added mineral/vitamin mix ^3^. Over the last decade these products have been widely used and have achieved impressive results in community-based therapeutic care (CTC) programmes ^4, 5^. Although a commercial product is available from Nutriset (Plumpynut) the hope was these recipes could be adapted for local manufacture and tested in field trials ^6^.

Whilst there have been significant developments in CTC for children with SAM, children hospitalised with severe and complicated acute malnutrition continue to experience poor outcomes with inpatient mortality rates of 20%, despite implementation of WHO guidelines ^7^. Whilst the feeds result in nutritional (anthropometric) recovery for the 80% who survive admission, this benchmark poorly predicts long term outcomes including death and/or re-hospitalisation over 12 months ^5^. Diarrhoea affects a large proportion of fatal SAM cases ^8^ with outcome substantially worse in those with diarrhoea (19%) than those without (9%). In addition, bacteraemia appears to be the major risk factor for mortality in SAM cases complicated by diarrhoea ^8^. A prospective study investigating associations between diarrhoea, enteropathogens, and markers of systemic and intestinal inflammation with mortality in Malawian children hospitalised with SAM found that high faecal calprotectin, low levels of faecal short-chain fatty acids (SCFAs) and markers of systemic inflammation were significantly associated with mortality ^9^. Multiple lines of evidence indicate that several domains of gut function are aberrant in children with SAM. Intestinal atrophy ^6^ in SM results in functional loss of brush border disaccharidases (lactase, maltase and sucrase) ^7, 10^ which exacerbate diarrhoea and impair recovery. Third, there is a significant gut microbiota immaturity ^11^, depletion in obligate anaerobes leading to less efficient nutrient utilization and high levels of pathogenic flora in children with SM which are only partially ameliorated following three weeks of standard nutritional interventions ^12^.

Current treatments and therapeutic feeds do not address all of these mechanisms and may exacerbate others. For example, the main carbohydrates in F75 and F100 are the disaccharides maltodextrin, sucrose and lactose, which are normally hydrolysed by brush border disaccharidases. However, intestinal atrophy ^13, 14^ and resultant functional lactase, maltase and sucrase deficiency ^15, 16^ have been demonstrated in SAM. High concentrations of undigested lactose in the intestinal lumen can result in osmotic diarrhoea, disrupting recovery ^17, 18^.

We propose that intestinal mucosal integrity, intestinal function, and gut microbial diversity can be restored ^19^ by providing substrates that induce saccharolytic fermentation in the gastrointestinal tract ^20, 21^. Legumes, rich in fermentable carbohydrates, improve gut microbial diversity and lead to production of short chain fatty acids which improve immunological and metabolic function of the gut in other disease settings ^16, 17^. A landmark study in Malawi, showed that while F75 and F100 supported weight gain (the primary measure of successful 'nutritional' rehabilitation), the gut microbiota did not recover ^22^. Addition of fermentable carbohydrates (most commonly fructo- and galacto-oligosaccharides) to formula milks have been successful in increasing the concentrations of faecal Bifidobacteria and reducing stool pH in healthy children ^23^. However, this has not been translated to the formulae used in children with SAM in low- and middle-income countries where case fatality remains a major problem ^24^. Our research group have developed and tested in a Phase II trials legume-based formulae for children with SM aimed at optimising gut function and ‘feeding the microbiome’, which has provided early indications of improved gut health and better clinical outcomes ^25, 26^.

We describe the development process of a lactose-free, fermentable carbohydrate-containing (chick-pea) alternative to traditional F75 and F100 formulae for the treatment of SAM, for use in a phase II controlled clinical trial in Uganda: Modifying Intestinal MicroBiome using Legume-based feeds (MIMBLE II) ^25^. 

## Methods

### Target nutritional profile

For the novel feed developed here in April 2018 (Campden BRI, Chipping Campden, UK) it was decided to match the nutrient profile of F100 formula, so that with specific feeding protocols to match energy and carbohydrate provision in both phases, the feed could be used for both stabilisation and rehabilitation. In addition to the target nutritional profile, the current product was developed with two additional specifications as outlined above: to remove lactose as a disaccharide source and to ensure the final product had a resistant starch content of 0.4–0.5%.

WHO provides recipes for F75 and F100 formulae, so they can be produced with basic ingredients that are widely available, summarised along with nutrition profiles in Table 1. In developing the current feed the philosophy behind the development of the feed was to aim to use components that were widely and cheaply available as of utmost importance, as it is designed for use in Africa. If we were able to demonstrate that the feed was having the desired physiological effects on the microbiome then the next step would be to consider a development in country using available food sources to replicate the novel formula.

**Table 1.** Recommended composition of nutritional milks for children with severe malnutrition.

Ingredient	F-75 (starter)	F-100 (catch up)
Dried skimmed milk *	25	80
Sugar (g)	70	50
Vegetable oil (ml)	35	70
Electrolyte/Mineral mix (ml)	20	20
Water to make up	1000ml	1000ml
Contents per 100ml		
Energy (kcal)	75	100
Protein (g)	0.9	2.9
Lactose (g)	1.3	4.2
Sodium (mmol)	0.6	1.9
Potassium (mmol)	4.0	6.3
Magnesium (mmol)	0.43	0.73
Zinc (mg)	2.0	2.3
Copper (mg)	0.25	0.25
% energy from protein	5	12
% energy from fat	36	53
Osmolarity (mOsmol/l)	413	419

* alternative recipes which use full-cream dried millk or liquid milk are: **F75:** full-cream dried milk 35 g, 100 g sugar, 20 g (or ml) oil, 20 ml electrolyte/ mineral solution, and make up to 1000 ml *or* full-cream cow’s milk (fresh or long-life) 300 ml, 100 g sugar, 20ml oil, 20 ml electrolyte/mineral solution and make up to 1000 ml **F100:** full-cream dried milk 110 g, 50 g sugar, 30 ml oil, 20 ml electrolyte/mineral solution, and make up to 1000 ml or full-cream cow’s milk (fresh or long life) 880 ml, 75 g sugar, 20 g (or ml) of oil, 20 ml electrolyte/mineral solution and make up to 1000 ml

The concentration of the feed was matched to F75 and F100 as appropriate for the stage of treatment [2] by the provision of Therapeutic CMV (Nutriset, Fr) at the point of feeding. The ingredients for the feed included, chickpea flour (legume/carbohydrate source), sucrose (carbohydrate source) lactose-free skimmed milk powder (protein source) and rapeseed oil (vegetable oil). Owing to the complexities of generating an infant milk feed for water reconstitution the feed was provided as a paste. The micronutrient (electrolyte and mineral mix recommended by WHO) was not included in the paste feed at the point of manufacture but during the trial, was added the feed at the time of administration using Therapeutic CMV (Nutriset, Fr)

### Nutritional profile determination

For this study, in order to reduced the cost of bulk-transporting the paste-based feeds to African we provided concentrates of the F100 formula (1 kcal/ml) by creating 4kcal/ml (F400), 3kcal/ml (F300) and 2kcal/ml (F200) recipes to improve the transportability of the feed. Each recipe would be suitable for the proposed feeding protocol as a single pack could be opened and fed to multiple patients in varying amounts according to an age- and weight-appropriate protocol using a dedicated SOP for feed reconstitution. For packing purposes all constitients of the recipe for F100 were multiplied by two, three or four, respectively, for F200, F300, F400 and a recipe created to meet these targets. We provided the clinical teams with a weight-based feed volume look-up table to faciliatate calculate how much the F200-F400 feed to provide as the equivalent to the relative requirement ie F75 (0.75 kcal/ml) and F100 (1 kcal/ml) feeds. Nutritional content analysis was performed by Campden BRI, using United Kingdom Accreditation Service (UKAS) accredited methods for energy, fat, carbohydrate, fibre and protein. Resistant starch measurement was completed by Eurofins, following published methods ^27^.

### Sourcing of ingredients, safety testing & relevant legislation

The risks and requirements that needed to be mitigated and met for each ingredient and finished product were conceptualised in 4 groups: *nutritional*, *microbiological, physical* and *chemical* (mycotoxins, heavy metals and pesticides). These were controlled through specifications at source by certificates of analysis (COA), but also with a supporting Hazard Analysis and Critical Control Point (HACCP) plan during process and then validated in a predetermined and detailed sampling plan in the finished product. The HACCP plan developed as per Gaze ^28^. During the process of feed development the plan covered biological (pathogenic bacteria, yeasts and moulds), chemical (residues of auxiliary production substances, acrylamide, allergens) and physical (glass and glass like substances, hard plastic, metal, wood, pieces of packaging, stones) hazards covering the from the time of the receipt of the raw materials to the eventual despatch of the labelled products.

With respect to the regulations where legumes were absent or not specified, guidance for cereals was adopted. Heat treatment would have no impact on heavy metals/pesticides and these also need to be controlled at source. These standards were collated ( Table 2) and ingredients were validated as above.

**Table 2.** Recipes and macro-nutrient profiles of concentrated feeds prior to retort and pre-gelatinisation.

Ingredients (in g/100g)	Legume- enriched double concentrated
Skimmed milk powder	7.2
Rapeseed oil	11.5
Legume flour	10.0
Sugar	9.0
Water	62.3

**Table:** 

Nutritional content per 100g *	Target	Pre-retort batch	Batch 1	Batch 2	Batch 3	Batch 4
Energy (kJ)	837	846.1	816	808	795	770
Energy (kcal)	200	203.2	195	193	190	184
Fat (g)	12	12.1	12.1	12.0	11.1	11.9
of which saturates (g)	-	0.9	-	-	-	-
Carbohydrates (g)	18	18.1	18.1	18.2	19.1	15.6
of which sugars (g)	-	12.0	-	-	-	-
Fibre, AOAC (g)	-	0.9	-	-	-	-
Protein (g)	5.6	5.6	5.8	5.9	5.9	5.3
Resistant starch (g)	0.4-0.5	0.09	0.4	0.4	0.3	0.3
Additional criteria **	Limit					
Acrylamide (µg/kg)	(<40µg/kg)	NA †	39	33	22	33
Lectin (HAU/g)	(<400HAU/g)	NA †	<40	<40	<40	<40
Tin (mg/kg)	(<50mg/kg)	NA †	0.02	0.01	0.02	0.01
Microbiological results ††	Standard					
pH (mean ± SD)	-	NA	5.65±0.03	5.70±0.02	5.70±0.03	5.81±0.01
Appearance (n/n acceptable)	Acceptable	NA	5/5	5/5	5/5	5/5
Odour (n/n acceptable)	Acceptable	NA	5/5	5/5	5/5	5/5
Microscopy (n/n negative)	Negative	NA	5/5	5/5	5/5	5/5
Cultures at 25°C (n/n no growth)	No growth	NA	5/5	5/5	5/5	5/5
Cultures at 37°C (n/n no growth)	No growth	NA	5/5	5/5	5/5	5/5
Cultures at 55°C (n/n no growth)	No growth	NA	5/5	5/5	5/5	5/5

Abbreviations: AOAC, Association of Official Agricultural Chemists; HAU, hemagglutinin units; SD, standard deviation * Nutritional content analysis was performed on 1 can per batch, resistant starch analysed in 2 cans per batch ** Acrylamide, lectin and tin testing completed for 1 can per batch †Acrylamide, lectin and tin not assessed in trial, pre-retort batch †† microbiological safety testing was conducted on 1% (5/500) cans per batch, but not on the pre-retort batch as it was not intended for use

To meet the criteria set by Commission Regulation (EC) 2073/2005 ^29^ on microbiological criteria for foodstuffs, five cans of each production batch were subjected to a full sterility test following pre-incubation at 30°C for 14 days (similar to ambient storage conditions in Uganda), and an incubation on different culture media (Liver Broth, Dextrose Tryptone Broth, Plate Count Agar and Eugon agar plus 1% Starch) at 25°C, 37°C and 55°C for a further 7 days. pH testing of final product performed by Campden BRI using United Kingdom Accreditation Service (UKAS) accredited pH meter in-house method (TES-AC-223). Acrylamide is a contaminant as defined in Council Regulation (EEC) No 315/93 2 and as such, it is a chemical hazard in the food chain. The European Commission Regulation (EU) 2017/2158 ^30^ has establishes a benchmark level of acrylamide content for baby foods of 40 μg/kg, which we adopted for this feed. Benchmark levels are not maximum residue limits but effectively critical limits to determine the efficacy or inefficacy of the processing systems to mitigate acrylamide formation. Finally, as per Commission Regulation (EC) No 1881/2006 ^31^, the legume-based feed was tested for tin and lectin which respectively needed to be lower than 50 mg/kg and 400 HAU/g. Acrylamide was analysed using liquid chromatography-tandem mass spectrometry (LC-MS/MS), lectin concentration by hemagglutination assay, and tin content by inductively coupled plasma – mass spectrometry (ICP-MS), all undertaken by Campden BRI (Chipping Campden, UK). Batch-by-batch results are summarised in the Extended data ^32^.

### Processing of ingredients, testing of processes and final processing pathway

After homogenising the dry ingredients in a Giusti Vesuvio cook/cool system, the oil and water were incorporated, and an oil-in-water emulsion created. Cans with standard Epoxy lacquer (73mmx62mm, from PromoCan Ltd) were filled with the feed using a can depositor to a fill weight of approximately 205g and seamed prior to retorting (Carnaud metalbox MB6 can seamer, Bead vertical 3 crate retort). One container was used for measurement of heat penetration and this was located in the centre of each layer in the retort to control the temperature and ensure that the cans had been processed at 121.1°C to achieve a F _o_ for =20 minutes. The canned feed was then had to be processed in the retort within two hours after its temperature had fallen below 63°C to prevent the growth of microorganisms.

## Results

### Ingredient contaminant testing

Table 3 presents the results of contaminant testing of the ingredients, namely rapeseed oil, chickpea flour and lactose-free skimmed milk powder. In short, all parameters met the previously outlined criteria for each ingredient for metals, pesticide residue, toxins and microbiological contamination. The COAs for ingredients demonstrated that the pesticides and heavy metals were within specification. 

**Table 3.** Chemical contaminants specifications for the ingredients.

	Criteria	Requirement	Result
Rapeseed oil	Lead	0.1 mg/kg (wet weight)	<0.005mg/kg
	Sum of dioxins (WHO-PCDD/F-TEQ)	0.75 pg/g fat	0.106pg/g
	Sum of dioxins and dioxin-like (WHO-PCDD/F-TEQ)	1.25 pg/g fat	0.116pg/g
	Sum of PCB28, PCB52, PCB101, PCB138, PCB153 and PCB180 (ICES – 6)	40 ng/g fat	0.0404ng/g
	Benzo(a)pyrene	2.0 µg/kg	0.809µg/kg
	Sum of benzo(a)pyrene, benz(a)anthracene, benzo(b)fluoranthene and chrysene	10.0 µg/kg	4.414µg/kg
	Erucic acid	50 g/kg	4g/kg
Chickpea flour	Lead	0.2 mg/kg (wet weight)	<0.005mg/kg
	Cadmium	0.05 mg/kg	0.012mg/kg
	Aflatoxin B1	2.0 µg/kg	<1.0µg/kg
	Aflatoxins B1, B2, G1, G2	4.0 µg/kg	<1.0µg/kg
	Ochratoxin A	3.0 µg/kg	<1.0µg/kg
	Pesticide residues	Any Haloxyfop <0.15mg/kg	Haloxyfop only 0.04mg/kg
Lactose-free skimmed milk powder	Aflatoxin M1	0.05 µg/kg	<0.03µg/kg
	Lead	0.1 mg/kg (wet weight)	0.02mg/kg
	Sum of dioxins (WHO-PCDD/F-TEQ)	2.5 pg/g fat	0.0253pg/g
	Sum of dioxins and dioxin-like PCBS (WHO-PCDD/F-PCB-TEQ)	5.5 pg/g fat	0.02759pg/g
	Sum of PCB28, PCB52, PCB101, PCB138, PCB153 and PCB180 (ICES – 6)	40 ng/g fat	0.0198ng/g
	Salmonella	absence in 25g	Absent
	Staphylococcal enterotoxins	absence in 25g	Not detected
	Enterobacteriaceae	10 cfu/g (5 samples)	<10cfu/g
	Coagulase positive staphylococci	10 cfu/g (5 samples of which 2 allowed up to 100 cfu/g)	<10cfu/g
	Melamine	2.5 mg/kg in food (except powdered infant formulae)	<0.05mg/kg

Table 2 also collates the specifications that need to be met for the ingredients with regard to chemical risks, as per COMMISSION REGULATION (EC) No 1881/2006 ^31^, and associated test results from COAs or testing carried out by Campden BRI. Certificates of analysis (COA) for the chickpea from two reputable suppliers were screened for presence of pesticides which were quantified and compared to Regulation (EC) No 396/2005 ^33^ on maximum residue levels of pesticides in or on food and feed of plant and animal origin, and Directive (EC) No 125/2006 ^34^ on processed cereal-based foods and baby foods for infants and young children. The Directive (EC) No 125/2006 ^34^ states that processed cereal based foods and baby foods must not contain residues of individual pesticides at levels exceeding 0.01 mg/kg, except for substances for which more specific levels have been set in Annex VI of the regulation.

Relevant COAs for lactose-free skimmed and whole milk powders were available and from a due diligence point of view, the ingredient was checked for the absence of melamine. Early options explored the feasibility of a fat powder but due to alignment on product format, a vegetable oil was chosen. A rapeseed oil was sourced with all relevant documentation. The refined oil used in the manufacture of this product is automatically sterilised in the final processing stage. Moreover, the product’s final composition precludes the growth of pathogens, spoilage organisms and virtually all other micro-organisms. Similarly, crystalline sucrose (granulated sugar) was sourced from a reputable supplier with all necessary documentation. An in-house source of de-ionised water was used to standardise batches and have controlled over mineral addition.

### Nutrition, microbiological and other safety testing

The final ingredients, target nutritional profile and nutritional, microbiological and final product safety testing results are presented in Table 3. Analyses on the raw cowpea flour revealed that it contained 0.9% of resistant starch which fell below the 3–4% expected, as reported in the literature ^35^. At an incorporation level of 10% of the legume feed into the legume feed, the F200 formula would only provide 0.09% of resistant starch. As a result, roughly 88% of the recipe would have to comprise of the flour in order to meet the requirements if the resistant starch levels remained unchanged during processing. Of the final 2000 cans produced in 4 batches (each of 500 × 200g cans), each were deemed to meet the target nutritional profile, albeit with some batch-to-batch variation. Pre-retort, the product contained 0.09% resistant starch, increasing to 0.3–0.4% following retort, likely due to retrogradation. Energy content was slightly below the desired target of 837kJ/100g, ranging from 770–816kJ/100g. Concerning macronutrients, fat content was within 0.1g/100g of the target (12g/100g) in 3/4 batches, while batch 3 contained 11.1g/100g. Carbohydrate content ranged from 15.6g/100g to 19.1g/100g, with a target of 18g/100g. Protein showed similarly low variability, ranging from 5.3–5.9g/100g, with a target of 5.6g/100g.

Acrylamide was below the limit in all batches (<40µg/kg), lectin concentration was below the detectable limits in all batches (<40HAU/g), well below the limit of <400HAU/g, and tin was similarly within the desired limit (<50mg/kg) in all batches. Following incubation at 25°C, 37 °C and 55 °C, of the 5 cans tested per batch, none were found to have any detectable growth of microorganisms, nor was there any observable organism with microscopy. Appearance and odour were both determined to be acceptable in all sampled cans. F200 achieved a F _o_=20 after 55 minutes, whereas within this same time the F300 had only achieved a F _o_= 7.62.

## Discussion

**Figure 1.**
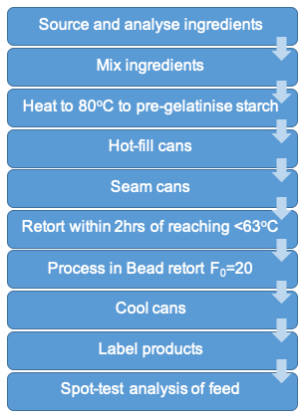
Final production process of legume-based feed.

The development processes described above are summarised in Figure 1. This approach resulted in a safe and palatable product, which met the target nutritional characteristics of double concentrated F100 to address shortcomings in current feeds used to treat SAM. Selecting a single nutrient profile based on double-concentrated F100 (termed F200), which was adapted for use in stabilisation and rehabilitation phases, ensured simple product use on site, and reduced chance of providing the incorrect formula. The fluid lost through concentration was accounted for in the feeding protocol by provision of additional oral fluid to match standard treatment. Otherwise children who required treatment for dehydration would be managed as per WHO guidelines, i.e. through oral rehydration solution (ReSoMal) and/or intravenous rehydration using Ringer’s lactate solution with 5% glucose [2].

With regards to raw materials, out of all the required ingredients, if we had opted for our preferred legume source, cowpea, this posed the biggest hazard risk as the biggest risk was cowpea flour which would need to comply with levels permitted for contaminants in foodstuffs intended for infant consumption. Despite thorough searching, it was not possible to find a suitable source of cowpea flour with the appropriate COA.We therefore opted for chickpeas as a legume source. Chick peas as also widely grown and eaten throughout Africa.

Despite the fact that ‘safe’ ingredients could be sourced, handling steps were unavoidable. A validation plan was created to extensively evaluate risks in the finished product. As not every product could be tested, a system allowing for a kill step and CCP (critical control point) at the end of any handling steps was adopted. Lectins and other anti-nutrients would need to be hydrolysed through heat treatment (e.g. retorting). Nevertheless, mycotoxins cannot be hydrolysed and must be prevented at the source. The feed could have taken the form of a dry mixed powder, later diluted and cooked in water; a low water activity fat-based paste which would have been sterilised; or a commercially sterilised product, where the organisms of concern would be controlled to a suitable level over the intended shelf life and storage conditions. We did not provide a shelf-life as this was a non-commercial product dedicated for a specific clinical trial with an intended short shelf life required.

In the absence of aseptic production, which is not widely available in lower to middle income countries (LMICs), various risks needed to be extensively evaluated. Ready-to-use low water activity (Aw) fat pastes already exist (e.g. Plumpy’Nut® Nutriset, Paris) and could easily be created for this study. However, it could not be sterilised through heat treatment due to its low moisture content. A low Aw fat paste was therefore not a viable option. The standard formulae recommended by WHO, F75 and F100, are dry powders which are mixed and heated in water prior to consumption. But similar issues to low Aw fat pastes occurred with a powdered product format, with the additional risk of validating homogeneity. The choice was therefore in favour of a water-based paste which could be commercially sterilised.

**Figure 2.**
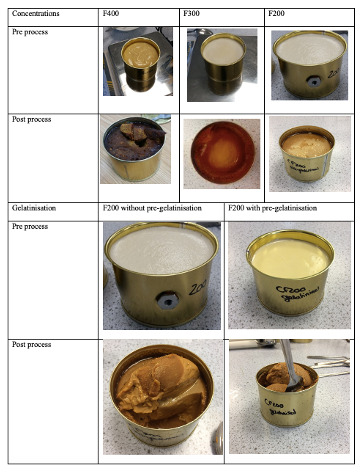
The effect of retort processing and pre-gelatinisation on texture of different feed concentrations.

It was recorded that during the process the texture of the feed after pre-gelatinisation of the starch was thicker before retorting than without pre-gelatinisation, and comparable to custard. After retorting, the texture was similar to mashed potatoes without sedimentation in the bottom of the cans which made it more homogeneous and suitable for a feeding trial ( Figure 2). In terms of palatability, the legume-based feed had legume and caramel flavours. If the texture of the products F200, F300 and F400 was very suitable before retorting, the texture of F400 became hard after retorting and made it unsuitable for a feeding protocol using a syringe. In contrast, the F300 and F200 retained adequate paste-like consistency, suitable for use. However, due to the difference in viscosity, the products heating behaviour was different. The F300 heated most likely via conduction and a lot slower than the F200 which heated via convection. Overall, due to shorter processing time and better viscosity, F200 was the most feasible. Another issue was that the feed formed a thick sediment in the bottom of the cans which made the feed inhomogeneous and unsuitable for a feeding trial. This issue was mitigated by heating the feed up to 80°C prior to filling in the sealed system with the vent closed to pre-gelatinise the starch and minimise the moisture loss.

Table 4 compares the macro- and micronutrient provision by standard feeds to the legume-enriched feed during stabilisation and rehabilitation phases, inclusive of additional fluid/micronutrient supplementation at the point of use for legume-enriched feed, showing close alignment. This demonstrates an equivalence in terms of nutrient provision, with the legume-enriched feed matching or exceeding the WHO-recommended feeds for macro- and micronutrient provision. Specific improvements include non-reliance on local sources of clean water, a significant reduction of disaccharide content, and addition of significant resistant starch content to directly support gut microbial populations. Challenges encountered included difficulty matching the micronutrient profiles F75/F100 in a ready-to-use product, which was ultimately addressed by correction at the point of feeding. Fluid intake also had to be specifically addressed in the final study protocol to account for fluid lost through concentration.

**Table 4.** Comparison of macronutrient and micronutrient provision during stabilisation and rehabilitation by standard feeds (F75/F100) and legume-enriched feed; data provided per kg of child weight per 24hrs.

		Stabilisation Phase			Rehabilitation Phase	
		Target[2]	F75	Legume-enriched	F100	Legume-enriched
Feed Delivery (kg ^-1^24hrs ^-1^)	Feed amount in 24hrs	-	135ml	50g	150ml	75g
	Additional Fluid (ml)	-	-	100	-	105
	Therapeutic CMV (g)	-	-	0.66	-	0.83
Macronutrients (kg ^-1^24hrs ^-1^)	Fluid from feed (ml)	-	135.0	131.1	150.0	151.7
	Energy (kcal)	100	101.3	101.6	150.0	152.4
	Energy (kJ)	420	425.3	426.7	630.0	640.1
	Protein (g)	1–2g	1.2	2.8	4.4	4.2
	Fat (g)	-	3.5	6.1	8.5	9.1
	Carbohydrate (g)	-	16.9	9.0	14.3	13.5
	Lactose (g)	-	1.8	<0.1	6.3	<0.1
	Resistant starch (g)	-	-	0.2	-	0.2
Micronutrients (kg ^-1^24hrs ^-1^)	Potassium (mmol)	4.00	4.86	6.78	8.85	10.17
	Sodium (mmol)	1.00	0.81	0.82	2.85	1.23
	Magnesium (mmol)	0.60	0.58	0.77	1.10	1.15
	Zinc (µmol)	30.00	50.63	64.19	54.00	96.29
	Copper (µmol)	4.50	5.83	9.89	7.20	14.84
	Vitamin A (RE mg)	0.15	0.41	0.31	0.45	0.47
	Thiamine (mg)	0.07	0.11	0.15	0.14	0.22
	Riboflavin (mg)	0.20	0.41	0.42	0.45	0.63
	Niacin (mg)	1.00	1.15	2.10	1.43	3.15
	Pantothenic acid (mg)	0.3	0.69	0.63	0.86	0.94
	Pyridoxine (mg)	0.07	0.14	0.15	0.15	0.22
	Biotin (mg)	0.01	<0.01	0.02	0.02	0.03
	Folic acid (µg)	100.00	45.90	73.49	57.00	110.24
	Vitamin B12 (µg)	0.10	0.41	0.21	0.45	0.31
	Vitamin C (mg)	10.00	11.48	21.00	14.25	31.50
	Vitamin D (µg)	3.00	6.62	6.30	8.55	9.45
	Vitamin E (mg)	2.20	6.62	4.62	8.55	6.93
	Vitamin K (µg)	4.00	11.07	8.40	14.25	12.60

Abbreviations: RE, retinol equivalents

### Future investigation

The safety and efficacy of the resultant product will be assessed in a phase II randomised controlled trial ^25^. Briefly, the MIMBLE II trial is an open-label controlled trial in 160 Ugandan children with SAM, defined by mid-upper arm circumference < 11.5cm and/or presence of kwashiorkor. The trial will be conducted Mbale Regional Referral Hospital nutritional rehabilitation unit. Children will be randomised on a 1:1 basis to the lactose-free, chickpea enriched feed containing 2kcal/ml, provided in quantities to match usual energy provision (experimental) or WHO standard treatment F75 (0.75kcal/ml) and F100 (1kcal/ml) feeds (control). The co-primary outcomes are change in MUAC at day 90 and survival to day 90. Secondary outcomes include moderate-to-good weight gain (>5g/kg/day), the development or worsening of diarrhoea (>3 loose stools/day) and time to diarrhoea resolution; time to oedema resolution (if the child presents with kwashiorkor (oedematous malnutrition)) and changes in intestinal biomarkers from admission to day 28 (faecal calprotectin). Supportive physiological data (to demonstrate whether the feed strategy is positively impacting gut function and microbial diversity) will include percentage changes in relative populations of gut microbiota, changes in generation of short chain fatty acid changes in host and microbiota metabolic products. In addition to the main trial outcomes we will assess palatability and safety of the nutritional feed.

Based on the findings of this 160-patient trial we may require additional refinements to the feed composition and adaptation of the processing and manufacturing methods from this work, so that a lactose-free, prebiotic feed can be quickly and safely manufactured in any developing country.

## Sponsor

Imperial College London is the main research Sponsor for the MIMBLE II clinical trial that this feed was designed for. For further information regarding the sponsorship conditions, please contact the Head of Regulatory Compliance at:

Joint Research Office, Room 221b, Medical School Building, St Mary’s Campus, Norfolk Place, London, W2 1PG. Telephone: +44 (0) 020 7594 1872.

The sponsor and funder played no role in the feed design, manuscript preparation and the decision to submit the report for publication.

## Data Availability

Imperial College Research Data Repository: Supplemental File MIMBLE Feed analysis. https://doi.org/10.14469/hpc/8337 ^32^. Data are available under the terms of the Creative Commons Zero "No rights reserved" data waiver (CC0 1.0 Public domain dedication).
