# Supplementary material for: Development of a legume-enriched feed for treatment of severe acute malnutrition
Source: Wellcome Open Res. 2023 Jan 27;6:206. Originally published 2021 Aug 11. [Version 2] doi: 10.12688/wellcomeopenres.16771.2 (PMC9971697; doi:10.12688/wellcomeopenres.16771.2)
Supplement: Peer review history

# Peer review: Reviewer response for version 2

**DOI:** 10.21956/wellcomeopenres.20794.r54543

**Published:** 2023-02-20

**Authors:** Sumathi Swaminathan

**Affiliations:** 1 Division of Nutrition, St John's Research Institute, Koramangala, Bangalore, India

The authors have revised the document based on the review. 

- The manuscript does describe the development of a food product for children in the treatment of severe acute malnutrition. Presumably the product will be tested for contaminants before the next phase. The concern is that once the product is stored in the tin can, it may not be immediately distributed to children resulting in a time lag. The product will need to be tested based on the duration of time it will take for the product to reach from the manufacturer to the target child. This safety needs to be established before the efficacy study is conducted. 
- Please make it clear whether this product is intended for treatment only during hospitalization during the stabilization and rehabilitation alone or if the product will be continued further. 
- Table 4 refers to only the quantity of protein not the quality. Quality of protein is evaluated using the protein digestibility corrected amino acid score (PDCAAS) or more recently by Digestible Indispensable Amino Acid Score (DIAAS). As skim milk powder has been added the quality should be good, but needs to be reported. Using the composition of ingredients these can be calculated by the authors or the manufacturer. 
- During the next phase the acceptability of this product among children with SAM needs to be reported. If rapeseed oil is not used in the community, it certainly needs to be checked out for acceptability before the efficacy study is conducted.
- The grammar in some sections need to be checked and corrected. 

Is the work clearly and accurately presented and does it cite the current literature?

No

If applicable, is the statistical analysis and its interpretation appropriate?

Not applicable

Are all the source data underlying the results available to ensure full reproducibility?

No source data required

Is the study design appropriate and is the work technically sound?

Partly

Are the conclusions drawn adequately supported by the results?

No

Are sufficient details of methods and analysis provided to allow replication by others?

Partly

Reviewer Expertise:

Nutrition

I confirm that I have read this submission and believe that I have an appropriate level of expertise to confirm that it is of an acceptable scientific standard.

# Peer review: Reviewer response for version 1

**DOI:** 10.21956/wellcomeopenres.18496.r45474

**Published:** 2021-10-07

**Authors:** Paluku Bahwere, Peter Akomo

**Affiliations:** 1 Centre of Research in Epidemiology, Biostatistics and Clinical Research, School of Public Health, Free University of Brussels, Brussels, Belgium; 2 Independent Consultant, Nairobi, Kenya

The project reported aims to develop an alternative formulation to F75 and F100 that according to the authors will be superior in reducing mortality through eliminating lactose in the food hence reducing risk of osmotic diarrhoea and will better promote the growth of “good” intestinal bacteria commensal flora. This makes scientific sense, and the research team has provided sufficient scientific information to back its hypothesis. Our main concern is the lack of information on the proportion of malnourished children who will benefit from the research products if the results are positive. In our opinion the research come in too late as currently over 90% of severely malnourished children accessing treatment are treated as outpatient. Also, life-threatening osmotic diarrhoea occurs only in a small proportion of children requiring inpatient stabilisation, except in few countries such us Zambia. Whereas complete removal of lactose from the formula could be achieved and can procure some health benefits, it might worth also recognising some importance of lactose in infant nutrition including some level of microbiota-modulating effect.

 Some statements need to be corrected. The standard RUTF was not developed by Valid International. Also, F175 and F100 are not for treating uncomplicated SAM but for complicated SAM.

 It will be helpful to provide more details on the calculation of the sample size of the follow up. We are not sure that a sample size  of 160 will have sufficient power to demonstrate difference in mortality.

 For programmatic use, changes in rheological characteristics during storage are important aspects to be sure about. There is no mention of studying this aspect. It might to be good to include this phase and provide data on effect of storage

 Information on the cost will be very important for the uptake of the novel feed. Including some cost information is important even if it is limited to ingredients cost.

Is the work clearly and accurately presented and does it cite the current literature?

Yes

If applicable, is the statistical analysis and its interpretation appropriate?

Not applicable

Are all the source data underlying the results available to ensure full reproducibility?

No source data required

Is the study design appropriate and is the work technically sound?

Yes

Are the conclusions drawn adequately supported by the results?

Yes

Are sufficient details of methods and analysis provided to allow replication by others?

Yes

Reviewer Expertise:

Undernutrition

We confirm that we have read this submission and believe that we have an appropriate level of expertise to confirm that it is of an acceptable scientific standard.

# Peer review: Reviewer response for version 1

**DOI:** 10.21956/wellcomeopenres.18496.r45404

**Published:** 2021-08-25

**Authors:** Sumathi Swaminathan

**Affiliations:** 1 Division of Nutrition, St John's Research Institute, Koramangala, Bangalore, India

The authors describe the process of development of a legume-based food product designed to improve the gut microbiome diversity and integrity and to reduce the rate of diarrhoea in hospitalized children with severe acute malnutrition. The results of this group’s pilot clinical trial is reported in the journal Cell Reports (Calder *et al.*, 2021). ^1^

 The description of the development of the formulation seems complicated with proper justification for the choice of ingredients and the type of analysis done not provided. The laboratory analysis seems to have been done for nutrients, microbial content, pesticide, environmental pollutants, heavy metals, and adulterants for only the individual ingredients and not the finished product. It appears that the shelf-life test was not done as it is not reported. A product's shelf life needs to be reported along with organoleptic and nutrient stability over a period along with absence of microbial growth.

 The quality of protein has not been evaluated for this food product. This is an essential requirement in the development of a food product particularly in children with SAM.

 Specific comments on the manuscript are as follows:

 Introduction: The authors mention Southeast Asia and sub-Saharan Africa, when, it is Asia and sub-Saharan Africa. Please refer to   UNICEF-WHO-The World Bank: Joint child malnutrition estimates — levels and trends – 2021 edition.The introduction needs can be more focussed beginning with the burden, the current solution, the reason development of the legume-based product giving a brief paragraph on diarrhoea, and the gut microbiome and the aim of the current study. Several sentences can be shifted to the discussion section.

 The target nutritional profile section in the methods is difficult to comprehend. It mentions “match the nutrient profile of F100 formula, so that with specific feeding protocols to match energy and carbohydrate provision in both phases, the feed could be used for both stabilisation and rehabilitation”. The meaning of this part of the sentence is not clear. Further, the next paragraph mentions that the micronutrient content was matched for F75 or F100.  To add to the confusion, “Micronutrient concentration of the feed was matched to F75 and F100 as appropriate for the stage of treatment [2] by the provision of Therapeutic CMV (Nutriset, Fr) at the point of feeding. The meaning of this is again difficult to understand in the context of the previous section written.

 The authors need to justify the need to concentrate the F100 formula (1 kcal/ml) by creating 4kcal/ml (F400), 3kcal/ml (F300) and 2kcal/ml (F200) recipes. Presume this was done by differential addition of water. Or was this done by a different method?

 In the section on Sourcing of ingredients, safety testing & relevant legislation a lot of jargon has been used.  The meaning of this statement “With respect to the regulations where legumes were absent or not specified, guidance for cereals was adopted” is not clear. The paragraph starting with “To meet the criteria set by Commission Regulation------ is undecipherable and its relevance is not clear.

 A tin container seems to have been used. This cannot be cost-effective in a country like Uganda. If tin was used there can be traces of metals leaching into the food product. The tin content has been analysed for each batch and the content is low, but not done during a shelf-life test.

 The composition of the food product includes rapeseed oil. This does not seem to be used in the Ugandan diet. The justification for the use of this oil, particularly as it contains erucic acid is required.

 There is no clarity on the type of legume used, that is, whether it was finally cowpea or chickpea. The manuscript published in the journal Cell Reports mentions cowpea. So which legume was used?

 Shelf-life studies need to be done before the product is used for a study. If it has been done it needs to be reported. The manuscript should then be modified once this is completed.

Is the work clearly and accurately presented and does it cite the current literature?

No

If applicable, is the statistical analysis and its interpretation appropriate?

Not applicable

Are all the source data underlying the results available to ensure full reproducibility?

No source data required

Is the study design appropriate and is the work technically sound?

Partly

Are the conclusions drawn adequately supported by the results?

No

Are sufficient details of methods and analysis provided to allow replication by others?

Partly

Reviewer Expertise:

Nutrition

I confirm that I have read this submission and believe that I have an appropriate level of expertise to confirm that it is of an acceptable scientific standard, however I have significant reservations, as outlined above.

## References

[1] : Modifying gut integrity and microbiome in children with severe acute malnutrition using legume-based feeds (MIMBLE): A pilot trial. *Cell Rep Med* .2021;2(5) : 10.1016/j.xcrm.2021.100280 100280 10.1016/j.xcrm.2021.100280 34095882PMC8149470
